# Assessing International Sport Federations' Sustainability Practices: Toward Integrating Sustainability in Their Main Sports Events

**DOI:** 10.3389/fspor.2021.752085

**Published:** 2022-01-13

**Authors:** Philjoo Moon, Emmanuel Bayle, Aurélien François

**Affiliations:** ^1^Institute of Sport Sciences, University of Lausanne, Lausanne, Switzerland; ^2^Laboratoire Cetaps, University of Rouen, Rouen, France

**Keywords:** sustainability, social responsibility, international sport federations, multiple case studies, neo-institutional theory

## Abstract

**Research Question:** Sustainability has become a pressing issue for a wide range of organizations, including sports' world governing bodies. This paper examines (1) how sustainability can be defined in the context of international sport federations and (2) how international federations implement social and environmental sustainability practices. We used an eight-dimensional analytical framework to analyze multiple case studies and drew on neo-institutional theory to interpret the recent changes international federations have made with regard to sustainability.

**Research Methods:** Our methodology combined a multiple case study with analyses of official documents and in-depth semi-structured interviews.

**Results and Findings:** Our six case studies revealed five approaches to sustainability: (a) implementing sustainability pilot events; (b) partnering with NGOs; (c) partnering with sustainability consultancies; (d) creating a sustainability committee; and (e) launching a comprehensive sustainability strategy with at least a full-time sustainability manager.

**Implications:** In terms of theory, examining our data through the lens of neo-institutional theory provides insights into international federations' recent sustainability behaviors. Our findings enabled us to draw up a “sustainability ladder” of sport federations' responsibilities, which can be used to assess the degree to which they have embraced sustainability. In practical terms, our findings should encourage more sport federations to take concrete steps to improve their sustainability by implementing the five approaches.

## Introduction

Since they first came into being at the beginning of the 20th century, international sport federations (hereafter IFs) have evolved from volunteer-run associations to highly professional organizations, the largest of which (e.g., FIFA) employ up to 700 staff (Clausen and Bayle, 2017). Although an IF's primary role is to develop its sport internationally (see Clausen and Bayle, 2017; Clausen et al., 2018), many IFs are becoming increasingly business-oriented and are commercializing their major events. For some IFs, organizing events has become a core activity and a true business involving numerous commercial partners, such as sponsors, broadcasters, and marketing agencies. At the same time, they are starting to pay more attention to sustainability issues.

Originally devised for profit-oriented enterprises, the notion of social responsibility and accountability has spread to non-profit organizations of all types and sizes, including non-profit sport organizations (Zeimers et al., 2019). In contrast to private companies, social responsibility has always been considered an implicit duty for IFs, due to their status as non-profit organizations. However, in recent years, a growing number of IFs have started engaging in explicit social responsibility activities, notably by showing a commitment to sustainability.

Indeed, the International Olympic Committee (IOC) began removing the term “social responsibility” from its strategy documents in the mid-2010's (Bayle, 2016; François et al., 2020), replacing it with “sustainability” (Bayle, 2016), and it now uses “sustainable development” as an umbrella term covering all its social responsibility initiatives. The IOC's substantial efforts to promote sustainability include launching, in 2016, its “IF Sustainability Projects” (today, “Sustainability Case Studies”), encouraging more IFs to organize their sports events in a sustainable manner, and drawing up an IOC sustainability strategy. In addition, it appointed a sustainability manager in early 2017 (IOC Sustainability Report, 2018) and published the “IOC Sustainability Essentials” guidebook in 2018.

Over the last decade, sustainability has become a key issue in the world of sport. Although efforts to apply the precepts of corporate social responsibility (CSR) to professional sport clubs or other national-level sport organizations have been widely researched (e.g., Babiak and Wolfe, 2006, 2009, 2013; Walters, 2009; Babiak, 2010; Walters and Tacon, 2010; Babiak and Trendafilova, 2011; Anagnostopoulos and Shilbury, 2013; Anagnostopoulos et al., 2014; François and Bayle, 2015: François and Bayle, 2017; Robertson et al., 2018; Zeimers et al., 2018, 2019, 2021; François et al., 2019; etc.), IFs' sustainability initiatives remain understudied. The present study helps fill this gap in the literature by exploring what sustainability means to IFs and how their approach to sustainability differs from the approaches used by corporations. To this end, we analyzed the ways IFs of different sizes and with different resources and capacities implement sustainability practices. We used our results to draw up a more precise definition of sustainability as it pertains to IFs. In this paper, we frequently refer to CSR, as this is the term most frequently used in the sport management literature. Nevertheless, CSR and sustainability are sometimes used interchangeably, and sometimes sustainability is used as a general term and CSR is used to describe the way in which organizations embrace sustainability.

Our study makes three contributions. First, it identifies five approaches to sustainability. Second, it throws light onto what IFs mean by sustainability. Third, by analyzing our dataset through the prism of neo-institutional theory, we provide a better understanding of why IFs have increased their sustainability efforts in recent years.

## Theoretical Background

### The Emergence of CSR in Sport

Because “talk of social responsibility” in sport originally referred to CSR, rather than sustainability, it is essential to review the emergence of CSR in sport. As the notion of CSR became more salient to businesses, sport organizations also started adopting socially responsible behaviors (Sheth and Babiak, 2010; Babiak and Trendafilova, 2011; Misener et al., 2013). However, sport did not start engaging with CSR to any significant extent until the early 1990's (Kott, 2005; Robinson, 2005; Babiak and Wolfe, 2013). Professional leagues (e.g., soccer, American football, baseball, basketball, ice hockey) were the first sport organizations to embrace the concept of CSR, and they rapidly began implementing a variety of CSR initiatives (Babiak and Wolfe, 2013). Over the last 30 years, the range of CSR-related initiatives undertaken by sport organizations has expanded to include philanthropy, community involvement, youth education, and youth health (Babiak and Wolfe, 2009; Walker and Kent, 2009; Paramio-Salcines et al., 2013).

Given the recent massive growth in the size of sporting events and the ambitions of rights holders, it has become vital to consider these events' environmental and social impacts (Turner et al., 2011). This realization led to the publication of ISO 26000, in 2010, to provide businesses and other organizations with guidance on how to operate in a socially responsible way^1^. Two years later, in conjunction with the London 2012 Olympic Games, the International Standards Organization published a separate set of standards (ISO 20121) for sustainable event management that, for example, provides guidelines on mapping the economic, environmental, and social impacts of sports events (García-Muñoz, 2013).

### Social Responsibility and International Sport Federations

IFs also have an obligation to act in socially responsible ways. Sports event organizers are coming under increasing pressure to consider their overall impacts on society and to implement CSR initiatives (Paramio-Salcines et al., 2013). Most IFs of Olympic sports were created in the late 19th century or early 20th century in order to produce standardized rules for their respective sports (Clausen, 2018). At this time, they were entirely amateur organizations (Inglis, 1997) that depended on volunteers and that did not seek financial income (Cornforth, 2001; Clausen, 2018). They have since become world governing bodies for their sports and are responsible for delivering top-class sports events, maintaining the integrity of their sport, and overseeing their affiliated continental and national federations, while meeting the needs of internal and external stakeholders, such as host cities, sponsors, and broadcasters (Jonker and De Witte, 2006; García-Muñoz, 2013). In commercial terms, they occupy an intermediate position between professional sport leagues and amateur national sport federations, most of which receive a large proportion of their annual budgets from government. In contrast to professional sport leagues, IFs are non-profit organizations which pursue income in order to ensure their financial independence.

This new environment has influenced the identity of IFs, which have become increasingly professional and commercial since the end of the 20th century. More specifically, the Olympic IFs started to realize the commercial value of their major events and of broadcasting and logo/naming rights, over which they have begun to assert ownership (Clausen and Bayle, 2017). According to Zeimers et al. (2021), this new operating environment has resulted in IFs becoming hybrid organizations whose sporting role is increasingly accompanied by commercial objectives, which they try to counterbalance by undertaking social initiatives. Nagel et al. (2015) identified four reasons why IFs have become more business-like and have begun facing questions about their CSR: (1) the growth of top-level international sport; (2) financial instability (business concerns); (3) the need to satisfy a greater range of stakeholders (e.g., numerous commercial partners); and (4) the need to adopt modern forms of communication. The resulting increase in commercialization has led to a concomitant increase in calls for IFs to demonstrate their social responsibility.

### Neo-Institutional Theory as a Theoretical Framework

The general public and other stakeholders are increasingly calling upon non-profit organizations to account for their actions (Morrison and Salipante, 2007). Most IFs are now what Bayle (2015) calls hybrid organizations in that they combine a social mission (e.g., development of their sports) with business objectives (e.g., event bidding, broadcasting rights) (Clausen, 2018). As such, they must try to make a profit while keeping their non-profit stakeholders satisfied. Given the need for IFs to ensure the legitimacy of their roles and the importance now accorded to social responsibility, often referred to as sustainability, we believe that neo-institutional theory provides the most appropriate framework for investigating IFs' recent embrace of sustainability.

Neo-institutional theory holds that the environment pressures organizations to conform with prevailing rules, requirements, and social norms. Since it emerged in the late 1970's (Meyer and Rowan, 1977; DiMaggio and Powell, 1983), the theory has become “one of the dominant organizational approaches” (Krücken et al., 2017, p. 1). According to Palthe (2014), neo-institutional theory is also a useful framework for explaining organizational change. DiMaggio and Powell (1983) identified three institutional forces that lead to isomorphism between organizations: (a) coercive pressure, which stems from political power exerted by the state; (b) mimetic pressure, which arises from the need to respond to uncertainty; and (c) normative pressure, which arises from the norms embedded in a profession or industry (DiMaggio and Powell, 1983; Scott, 2001; Appari et al., 2009). These three institutional pressures contribute to the varying ways in which organizations may interact and depend on other organizations within their environment, and in turn affect the direction of their decision making (Vos et al., 2011; Johnston, 2013).

Scott (2001) built on this work to determine the “three pillars of institutionalization”: regulative, normative, and cognitive. First, some components may change because they are forced to by company policy (have to change); others may change in response to changes in norms (ought to change); and yet others may change because they value the new order and therefore embrace it (want to change). Powell (2007) summarized Scott's idea as follows: “Each … pillar offers a different rationale for legitimacy, either by virtue of being legally sanctioned, morally authorized, or culturally supported. In essence, each element (regulative, normative, and cognitive) provides a basis for legitimacy—a condition reflecting accordance with rules or laws, normative pressure, or cultural alignment” (Scott, 1995; Palthe, 2014). Indeed, legitimacy is a key aspect of neo-institutional theory (see Meyer and Scott, 1983; Strittmatter, 2016, p. 424). Suchman (1995) defined legitimacy as “a generalized perception or assumption that the actions of an entity are desirable, proper, or appropriate within some socially constructed system of norms, values, beliefs, and definitions.”

Numerous studies in the sport management literature have applied neo-institutional theory (e.g., Kikulis et al., 1992; Slack and Hinings, 1994; Kikulis, 2000; O'Brien and Slack, 2004; Edwards et al., 2009; Clausen, 2018). For instance, Godfrey (2009) applied Scott's (2001) three pillars of institutionalization to sport, Babiak and Trendafilova (2011) used institutionalism to explain sport leagues' motives for engaging in CSR and environmental sustainability, Strittmatter (2016) applied institutionalism to the Norwegian Olympic Committee's attempt to legitimize its bid for the 2016 Winter Youth Olympic Games, and Zeimers et al. (2019) employed institutional theory, together with resource-based view theory, to examine non-profit sport organizations' motivations for socially responsible programs. Finally, McLeod et al. (2020) adopted institutionalism to study the drivers and barriers of governance convergence in Indian sport.

Studies using institutional theory have shown that sport organizations are motivated to engage in socially responsible behaviors by the desire to adapt to their environment and the need to prove their legitimacy. However, previous research has focused on professional sports leagues, national associations, regional clubs, and national Olympic committees, whose motivations may be different to those of IFs, the focus of the present study. For example, professional sports leagues might engage in CSR activities for risk management or PR purposes, whereas IFs use CSR to show leadership as the world governing body for their sport. Nevertheless, institutional theory is an appropriate theoretical framework for examining CSR by IFs for two main reasons: (1) IFs face increasing pressure to demonstrate their legitimacy with respect both to their initial mission of developing their sport and to their new mission of meeting the business objectives of their stakeholders, including their commercial partners; and (2) IFs tend to adapt to their external environments (e.g., expectations from worldwide fans, athletes, national federations, host communities, media, etc.), as there is no international law with which they must comply.

### Research Model

**Figure 1:**
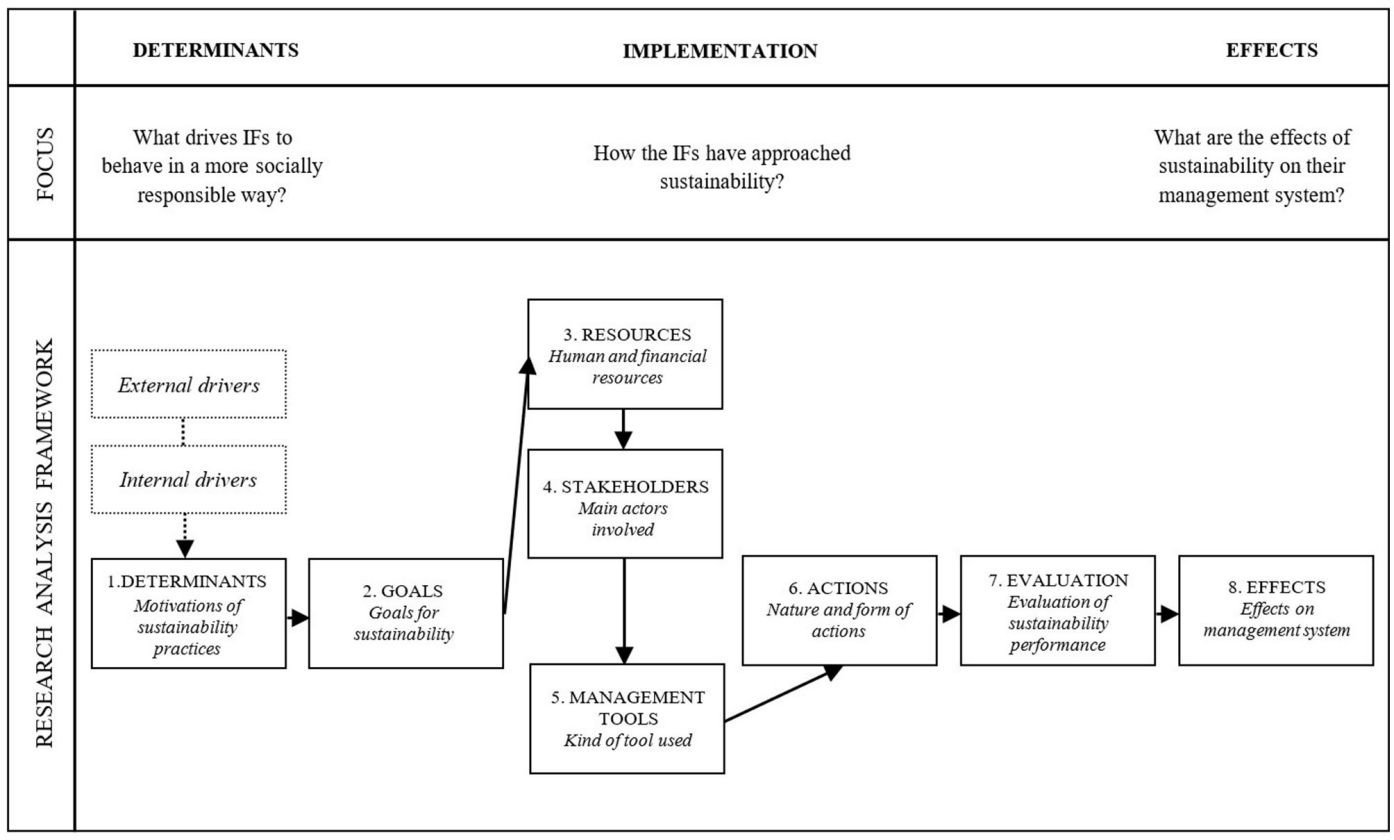
Analysis framework for the study (adapted from François and Bayle, 2015, p. 26).

For the present study, we used the analytical framework drawn up by François and Bayle (2015) to analyze the CSR practices of French professional sports clubs, to which we added an *effects* dimension to analyze the extent to which IFs embraced the concept of sustainability (see Figure 1). Organizations undertake social responsibility initiatives in response to external and internal factors. Once they have decided which initiatives to take, they set goals and secure the human and financial resources required. To implement these initiatives, they need to collaborate with their stakeholders and apply appropriate management tools. They must also cooperate with and motivate their internal staff. One of the critical factors in the process is evaluation for improvement (see Maon et al., 2009). Lastly, this whole process most likely affects their management system.

## Methods

Because IFs have only recently begun taking sustainability actions and because we expected different IFs to adopt different approaches to sustainability, we used a qualitative methodology involving multiple case studies, rather than focusing on a single case study.

### Case Selection

Given the paucity of research into CSR and sustainability actions by IFs, we began by screening the websites of Olympic IFs (28 summer Olympic IFs and 7 winter Olympic IFs) for relevant documents. We decided to focus on Olympic IFs, rather than on non-Olympic IFs, because the former tend to represent more popular sports and to have greater financial independence. We selected the IFs to include in our study by examining the 40 “IF Sustainability Projects” the IOC published in 2016 and 2017^2^ for three key factors.

First, so we could examine tangible sustainability actions, we looked for information about an Olympic IF's recent sustainability activities, in terms of the department, people (e.g., a designated sustainability manager), and/or committee involved. An important criterion was whether an IF had allocated human resources to concrete actions. For example, FIFA (soccer) and the IIHF (ice hockey) have actively implemented social and environmental sustainability initiatives via a specific Sustainability & Diversity Department (FIFA) or Environmental & Social Activities Committee (IIHF). These were the first two IFs selected for our case studies. Second, we wanted to include IFs of different sizes (small, medium, and large) in order to determine whether approaches to sustainability were linked to an IF's size. Third, we selected participants on the basis of their familiarity with and involvement in their organization's sustainability initiatives (Babiak and Wolfe, 2009). We chose key individuals involved in managing sustainable sporting events by examining lists of attendees at international sport and sustainability seminars (e.g., a seminar on “How can IFs prioritize environmental sustainability with their events?” held by the MSI^3^ in Lausanne, Switzerland, in October 2019; a workshop entitled “Sport and biodiversity: how can we build a stronger link?” held by the International Union for Conservation of Nature, in Gland, Switzerland, in April 2018, etc.).

Based on these criteria and our analysis of organizational documents, we selected nine Olympic IFs for interviews: the FEI (equestrian sports), FIFA (soccer), FIS (skiing), FISA (rowing), ICF (canoeing), IIHF (ice hockey), UCI (cycling), WS (sailing), and WT (taekwondo). As the next step, we conducted in-depth semi-structured interviews with key people at these IFs. The person we had arranged to meet at the UCI withdrew from the interview and we excluded the FIS and WT from our case studies following the interviews due to a lack of information. Nevertheless, the information gained during these two interviews shed light onto the recent embrace of sustainability by IFs. Hence, we conducted detailed case studies of the following six Olympic IFs: the FEI, FIFA, FISA, the ICF, the IIHF, and WS (see Table 1 below).

**Table 1:** IFs selected for case studies.

IF^*^	**Formation**	**Headquarters**	**NFs**	Number of staff^**^	Size^***^	Revenue^****^
			**(2019)**	**(2018–2019)**		**(2016)**
FEI	1921	Lausanne	134	98	L	CHF 54
FIFA	1904	Zurich	211	600–700	XXL	CHF 462
FISA	1892	Lausanne	155	21	S	CHF 6.6
ICF	1946	Lausanne	167	10	S	CHF 13.6
IIHF	1908	Zurich	76	32	M	CHF 30.4
WS	1907	London	144	27	M	CHF 19

* *FEI, International Federation for Equestrian Sports; FIFA, International Federation of Football Association; FISA, World Rowing Federation; ICF, International Canoe Federation; IIHF, International Ice Hockey Federation; WS, World Sailing*.

** *Source: each IF's website and interviews*.

*** *We determined size on the basis of the number of staff and annual revenue*.

**** *Shown in million Swiss francs. Source: FEI Financial Report 2017; FIFA Financial Report 2017; FISA Financial Audit Report 2016; ICF Financial Audit Report 2016; IIHF Annual Report (July 2017-June 2018); World Sailing Limited Report and Financial Statements (December 2017)*.

Of these six IFs, five represent summer Olympic sports and one represents a winter Olympic sport. They are all based either in Switzerland (Lausanne or Zurich) or in the UK (London). FISA is the oldest of the six IFs (founded in 1892); the ICF is the youngest (founded in 1946). They cover the full range of sizes of Olympic IFs as they employ between 10 (ICF) and 700 (FIFA) staff. Even though it has fewer staff than FISA, the ICF's income for 2016 (CHF 13.6 million) was higher than FISA's income because the ICF earns a large proportion of its income from the Olympic Games and 2016 was an Olympic year for the summer IFs. In non-Olympic years, the ICF's income is lower than FISA's. Consequently, both the ICF and FISA can be considered small Olympic IFs.

### Data Collection

We used two methods to collect data. We began by analyzing documents produced by IFs, in order to gain a general understanding of the concept of sustainability in relation to IFs and to select the cases we would study in detail. We did this by screening the Olympic IFs' websites and reviewing academic papers, book chapters, dissertations, sports magazines, IF annual reports and financial statements, CSR and sustainability reports, press releases, statutes, mission statements, organization charts, and other public and non-public documents produced by IFs, the IOC, Olympic Games organizing committees, and United Nations agencies, etc. As a second step, we conducted in-depth semi-structured interviews with people in charge of IFs' sustainability programs and with individuals who are familiar with the dynamics of sustainability in the context of international sporting events.

We obtained academic documents (online and offline) from the University of Lausanne's and the Olympic Studies Centre's (Lausanne, Switzerland) libraries, via Google Scholar or ResearchGate, or from the International Academy of Sport Science and Technology (AISTS) (e.g., research papers, team-project reports). The AISTS's Sustainable Sport and Event Toolkit System provided a further 50 documents describing environmental, CSR, and sustainability guidelines and good practices. Finally, we searched sports news websites (e.g., www.sportanddev.org, www.insidethegames.biz) for relevant information. These documents provided a useful overview of IFs' relationships with sustainability, but they did not answer fundamental questions concerning IFs' motivations for undertaking sustainability actions, their goals for these actions, the human and financial resources they allocate to sustainability, and the identities of their main stakeholders.

To address these questions, we conducted in-depth semi-structured interview with key personnel within our six selected IFs. The interview questions covered all eight dimensions of our analysis framework. The main questions we asked were as follows:

- ***Determinants***: What are your federation's motivations for engaging in sustainability actions?
- ***Goals***: What are your federation's short-term and long-term goals for sustainability?
- ***Resources***: What resources (e.g., human, financial) are allocated to sustainability actions? How does your federation fund its sustainability actions?
- ***Stakeholders***: Who are their main stakeholders and partners?
- ***Management tools***: What management tools does your federation use?
- ***Actions***: How does your federation implement sustainability programs? That is, how does it interact with internal staff and external stakeholders? What challenges has it faced? What does it do to overcome these challenges?
- ***Evaluation***: How does your federation evaluate its sustainability performance?
- ***Effects***: What are the effects of sustainability on your federation's management system? What is the impact of sustainability on its structure and/or management practices?

We began each interview by asking the interviewee for background information (e.g., how long they had worked for the IF; the number of staff in the organization). We then asked interview questions in order, but we generally adopted a discussion format based on open-ended questions.

In-depth semi-structured interviews allow researchers to collect open-ended data, to explore participants' thoughts, feelings, and beliefs about a topic, and to delve into personal and sometimes sensitive issues (DeJonckheere and Vaughn, 2019). Hence, semi-structured interviews can provide a better understanding of context (Easterby-Smith et al., 2008; Walters and Tacon, 2010) and enable researchers to follow an interview guide but to probe more deeply when necessary (Kvale and Brinkmann, 2009; Walters and Tacon, 2010). The interviews conducted for the present study provided the primary data we needed to answer the questions that were not answered by our document analysis. To increase data trustworthiness, where possible we conducted the interviews face-to-face, rather than by telephone, videoconferencing, or email (see Table 2).

**Table 2:** List of interviewees.

Organization^*^	**Size**	**Interviewee**	**Year**	**Duration (minutes)**	**Means**	**Place**	**Code**
FEI	L	Employee	2019	110	In person	Lausanne	P 1
FIFA	XXL	Employee	2019	76	In person	Zurich	P 2
FISA	S	Employee	2018	60	In person	Lausanne	P 3
ICF	S	Employee	2019	85	In person	Lausanne	P 4
IIHF	M	Employee + Committee member	2019	76	In person	Zurich	P 5
WS	M	Employee	2019	81	Skype	-	P 6
FIS	L	Employee	2018	62	Skype	-	P 7
WT	M	Employee	2018	68	In person	Lausanne	P 8
IOC		Employee	2019	52	In person	Lausanne	P 9
ILO		Employee	2019	47	In person	Geneva	P 10
UNESCO		Employee	2019	58	In person	Paris	P 11

* *FEI, International Federation for Equestrian Sports; FIFA, International Federation of Association Football; FISA, World Rowing Federation; ICF, International Canoe Federation; IIHF, International Ice Hockey Federation; WS, World Sailing; FIS, International Ski Federation; WT, World Taekwondo; IOC, International Olympic Committee; ILO, International Labor Organization; UNESCO, United Nations Educational, Scientific and Cultural Organization*.

We conducted the interviews, all in English, between December 2018 and May 2019. Participants were directors (five interviewees), managers (six interviewees), or committee members (one interviewee) of their respective organizations. They had worked for their organizations for between 2 and 16 years (mean = ~8 years). To gain a deeper understanding of IFs' recent concerns with sustainability, we also interviewed representatives of three other organizations involved in international sport (IOC, UNESCO, ILO). However, the staff at the United Nations Environment Programme who we contacted declined to take part in the study. Because social responsibility is a sensitive topic for non-profit organizations, including some IFs (see Waters and Ott, 2014), in order to reduce the risk that executives would refuse our request and withdraw their organization from the study, we decided to interview only one person from each organization. Furthermore, the ways in which the IFs involved implement their sustainability actions may have evolved since we carried out our case studies, which were based on organizational documents produced between 2015 and 2019 and on interviews conducted between December 2018 and May 2019.

### Data Analysis

We began each interview by asking the interviewee for permission to record the interview, using a digital recorder. We listened to the recordings in order to check the content and then transcribed them for further analysis. The 11 interviews generated 140 pages of single-spaced text. As a first analysis stage, we read through the transcripts to check data saturation for the eight dimensions of our analysis framework. This stage also enabled us to ask the interviewees to clarify some of their statements and to provide additional sources (e.g., bid documents, evaluation tools). We then read the transcripts twice more and coded key words, phrases, and sentences relating to the eight dimensions of the analysis framework. Because the lead author did all the transcription work manually, we reviewed each interview at least four times to ensure the coding was accurate. We then re-analyzed the coded information in the light of our research questions.

To increase trustworthiness, we triangulated the results of the interview analyses with academic papers and data from the IFs' and IOC's websites (e.g., FIFA sustainability report, “IF sustainability projects”). To ensure the participants' anonymity, we provide only their affiliations, but not their names or positions. Although quantitative studies often refer to interviewees by pseudonyms, this strategy was not appropriate for our study because the participants are members of a very small community and could therefore be easily recognized (Orb et al., 2001, p. 95).

## Findings: Five Approaches to Sustainability

Our analysis of different sized Olympic IFs revealed five different approaches to sustainability, as outlined in Table 3. The following sections discuss the implications of each approach.

**Table 3:** Five approaches to sustainability.

**Type**	**Main feature**	Main actors [no.]^*^	**Case (*size*)**
Type 1	Launching sustainability pilot events	IF (an employee—part-time^**^), LOCs, host cities, NFs, AISTS	ICF (*S*)
Type 2	Partnering with NGOs	IF (cross-department collaboration^***^), LOCs, host cities, NFs, NGOs	FISA (*S*)
Type 3	Partnering with sustainability consultancies	IF (an employee – part-time^**^), LOCs, host cities, NFs, sustainability consultancies	FEI (*L*)
Type 4	Leadership by a sustainability committee	IF (“sustainability” committee [5]), LOCs, host cities, NFs	IIHF (*M*)
Type 5	Launching a comprehensive sustainability strategy	IF (sustainability manager [1], sustainability commission [8]), LOCs, host cities, NFs	WS (*M*)
		IF (sustainability department [10]), LOCs, host cities, NFs	FIFA (*XXL*)

* *Numbers in square brackets refer to the number of people assigned to sustainability practices*.

** *An employee—part-time means that an existing employee (e.g., a marketing manager) spends some of their time (e.g., 20–30%) on sustainability programs (status as of early 2019)*.

*** *Cross-department collaboration means there is no sustainability manager, but all departments work together to implement sustainability actions under the leadership of a senior manager (in this case, FISA's Sport Director, status in early 2019)*.

### Approach 1: Launching Sustainability Pilot Events (e.g., the ICF)

According to our interviewee, the ICF's decision to embrace sustainability was motivated by both internal and external factors. The internal factors are (1) leadership, (2) employees' interest in sustainability, and (3) a management strategy; the external factors are (a) the general public's expectations, (b) pressure from the IOC, and (c) the fact that other IFs have begun, to a greater or lesser extent, introducing sustainability actions. DiMaggio and Powell's (1983) institutional isomorphism process (coercive, normative, mimetic isomorphism) applies to the ICF. Because canoeing directly involves water, its position on sustainability has been influenced by normative pressure to protect water quality. Its sustainability initiatives have also been influenced by the IOC and the federation has felt pressure to follow the example set by other Olympic IFs. The following quote illustrates why we adopted a neo-institutional perspective:

I really think it's mix of everything. I would say… for us, for instance, the first thing is **the natural link with the sport** because it's **an outdoor sport with water**. And then, the normative **pressure from the IOC** as I told you before… for sure in our case, and personal interest as I told you. Image of the ICF… the image is important because it gives also a good image of our federation, and the interesting thing is also **what other IFs are doing**. For me, it's an important point, you know. For sure, every time we want to do better and better. [....] I think it's a positive competition (Interview with P4, 2019).

One of the most common challenges in implementing sustainability actions is limited financial and human resources. Another important element is an IF's expertise in sustainability issues. The ICF has attempted to take concrete sustainability actions by organizing sustainability pilot events. Despite allocating just one person to sustainability (who, since 2017, has spent ~30% of their time on sustainability), the ICF has made efforts to overcome these challenges by working closely with local organizing committees (LOCs), host cities, AISTS, and athletes (e.g., ambassador programs). Both our interviewee and the ICF's 2018 Sustainability Pilot Event Report declared its first sustainability pilot event—the 2018 Canoe Slalom World Cup, in Augsburg, Germany, to be a success.

AISTS provided support in planning and evaluating the pilot event, for which it used the GRI G4 and the AISTS Sustainable Sport & Events Toolkit (SSE Toolkit) management and evaluation tools. Although the ICF's ability to pursue sustainability projects is restricted by a lack of human resources and expertise (it has neither a full-time sustainability manager or guaranteed support from AISTS for future events), it shows that small IFs with limited resources and expertise can still set off on the sustainability journey. Hence, we believe that the ICF typifies an approach to sustainability that is particularly suited to small sport organizations.

### Approach 2: Partnering With NGOs (e.g., FISA)

FISA exemplifies the importance of relevant expertise in implementing sustainability projects, aimed in this case at environmental sustainability. As the following quote illustrates, FISA has secured expertise in environmental sustainability management by partnering with the World Wide Fund for Nature (WWF) and subsequently with other NGOs.

**Through our relationship with WWF**, we have kind of access to their expertise. I am not a sustainability expert. If I have a specific question, I would refer to either the IOC Sustainability Manager or to WWF, and now more recently the IUCN (International Union for Conservation of Nature) is following our World Heritage sites commitment. We have that link as well, so... we don't have anyone, but we have WWF and IOC to support us (Interview with P3, 2018).

In fact, the WWF has played a pivotal role in helping FISA implement sustainability initiatives, such as its Environmental Management System, and in drawing up environmental requirements for LOCs. The case of FISA shows that IFs must assign at least one member of staff to sustainability and obtain a degree of expertise in sustainability if it is to implement effective sustainability actions. Although FISA made its sport director responsible for sustainability a few years ago, it has not yet appointed a specific sustainability manager (source: interview with P3, 2018). Consequently, its ability to consistently follow up and monitor its sustainability programs is limited.

Nevertheless, FISA provides a model for another approach to sustainability, especially for the IFs of outdoor sports (e.g., watersports). Recent studies have noted that smaller organizations take different approaches to CSR, as they have fewer resources and less organizational capacity than larger organizations (Baumann-Pauly et al., 2013; Wickert et al., 2016; Zeimers et al., 2021). Despite their limited human resources (no sustainability manager or sustainability committee), small IFs such as FISA can develop sustainability strategies with support from one or more NGOs.

### Approach 3: Partnering With Sustainability Consultancies (e.g., FEI)

Some of our interviewees noted that embedding sustainability terms in bid requirements and contractual documents is a way for IFs to overcome their limited power to impose sustainability on LOCs. However, the FEI has adopted a different approach to sustainability. Our interviewee emphasized the importance of developing an evaluation toolkit and guidelines and of encouraging member federations and LOCs to take sustainability initiatives voluntarily. Regardless of their scope and number, these initiatives are acknowledged on the FEI Sustainability System's website. As our interviewee explained:

We have 4,000 international events, so… this is more than anyone else. The majority of these are FEI sanctioned events, but they are still under the FEI umbrella. [….] What we are trying to achieve with the sustainability policy is to enable this happening **voluntarily with a lot of easy information, tools, and materials provided at all the 4,000 events**, so… this is the difference. [….] It is not enforcing on the organizers. We don't want to say… do this and that. There are already so many needs… the requirements for the organizers. So, our philosophy is not like you should. Here are a lot that we can support you with. And then, we enable, you know. By acting as a leader in this movement on doing a good thing on sustainability (Interview with P1, 2019).

The FEI developed its sustainability strategy in collaboration with three different sustainability consultancies. It worked with Quantis to draw up the FEI Sustainability Handbook for Event Organizers (2014) and in 2015, it worked with SchweryCade to carry out an Environmental Sustainability Survey (source: IF Sustainability Project – Environmental Sustainability Survey, IOC, 2016). More recently, it worked with Interseroh to develop its own sustainability toolkit by applying the GRI framework:

Working with the consulting company, Interseroh with them… the first thing we were doing is adopting very equestrian sport specific indicators from the GRI list and G4 indicators. And then, choosing the scope, and then, now we would be done choosing the scope… you know, how we measure that, but now, we are at a point of writing the exact initiatives as I told you… how to reduce paper, and then, creating a framework around it (Interview with P1, 2019).

Hence, the FEI has implemented a plan to encourage LOCs to voluntarily carry out sustainability programs. Like the ICF, the FEI does not have a full-time sustainability manager, but its events classification and sustainability manager spends (in 2019) up to 30% of their time on sustainability issues. Although the FEI's website does not yet include its Sustainability System, the FEI exemplifies a third approach to sustainability.

### Approach 4: Creating a Sustainability Committee (e.g., IIHF)

The IIHF and WS are the only IFs in our sample to have set up a sustainability committee or commission. For an organization to create a specific committee to oversee sustainability initiatives, it must have: (1) sufficient human and financial resources, (2) a degree of expertise in sustainability, and (3) a senior management team that believes in the importance of sustainability. This is the case at the IIHF. Since it set up its Environmental & Social Activities Committee in 2013, the IIHF has more actively implemented sustainability practices and launched numerous social and environmental projects. According to the two interviewees from the IIHF, the time the committee's five members spend on sustainability issues is equivalent to one half-time post, although they are not based at the IIHF's headquarters:

**Employee:** “At the moment, 70%…” (laughs)

**Committee member:** “I would say, actually I would say, I mean… you also have to take the work into consideration that the Committee as well. So, I will actually put the number of say… 50%. If you take one employee, I would say the Committee which is outside still working for the IIHF plus the secretary for the Committee. I would say it's 50%. (Interview with P5, 2019).

In addition, the IIHF has allocated the committee a specific budget (subject to approval by the IIHF Congress) for sustainability actions (source: interview with P5, 2019). Consequently, a lack of human and financial resources is not a major obstacle to conducting sustainability actions, as is the case for most smaller IFs. What is more, the committee includes members both from within and from outside ice hockey, and they have a certain degree of expertise in social and environmental sustainability issues thanks to their long experience in organizing (inter)national sports events. Just as importantly, the committee is supported by the IIHF's senior managers, including the executive board, which has shown great enthusiasm for conducting sustainability actions. Nevertheless, the IIHF differs from IFs such as WS and FIFA, as it does not have a sustainability manager (e.g., WS) or a sustainability department (e.g., FIFA). Consequently, the IIHF illustrates a fourth approach to sustainability, led by a sustainability committee.

### Approach 5: Launching a Comprehensive Sustainability Strategy (e.g., FIFA and WS)

FIFA's and WS's approaches to sustainability were motivated by a combination of **internal and external factors**. In the case of FIFA, the external factors were (1) the general public's expectations, (2) host countries' attitudes toward sustainability, and (3) UN recommendations, whereas the internal factors were: (a) leadership from the president and secretary general and (b) employees' values (source: interview with P2, 2019). For WS the external factors were: (1) sailors' expectations, (2) IOC recommendations, and (3) UN recommendations; internal factors were (a) leadership from the CEO, (b) employees' interest and ideas, and (c) a management strategy (source: interview with P6, 2019). Their active sustainability practices can be interpreted from a neo-institutional perspective by highlighting the external influences, although there are also internal drivers, as the following quote shows:

“Well... there is certain **expectations from the general public**. It's very popular. TV is that we have... football is in everyone's mind and part, so... this is definitely one. There is also another external factor which is **the situation in the host countries** we are staging the events. For example, the fact that we are hosting the World Cup we signed. The World Cup will be hosted in Qatar… then, it's inevitable that you start talking about human rights. You start talking about labor rights because the labor conditions in the country in general were very bad. It is not just expectations from the public; it's the actual situation in the country where we are going” (Interview with P2, 2019).

Unlike the other four IFs in our sample, FIFA and WS have launched comprehensive sustainability strategies and have recruited one or more full-time staff sustainability in order to embed sustainability into their core business of organizing sports events. Although the two federations have things in common, there are a number of differences in their approaches to sustainability. FIFA's sustainability initiatives are conducted by its Sustainability & Diversity Department, which has 10 full-time staff (source: interview with P2, 2019), whereas WS has a full-time head of sustainability and an eight-member Sustainability Commission (source: interview with P6, 2019). Both IFs have adopted international standards, such as ISO 20121, the GRI standards, and the Greenhouse Gas Protocol, as management tools or reporting frameworks for their sports events:

“We try to use standards that are understood by everyone and compatible as well. We used **ISO 26000** before the existence of **ISO 20121** because for the 2014 FIFA World Cup Brazil, ISO 20121 didn't exist, so we used that as a standard. When 20121 was created by ISO as a sustainability management system for events, then we applied that very clearly” (Interview with P2, 2019).

Both federations have also included sustainability criteria in their bid documents, so LOCs have to meet certain sustainability requirements. FIFA starts “sustainability preparations” for its flagship event (the World Cup, the world's largest single-sport event) earlier than WS, as FIFA's Sustainability & Diversity Department starts preparing for the World Cup 5–8 years before the event, whereas WS starts preparing for its world championships, in cooperation with the LOC, 2–3 years before the event.

FIFA's organizational structure has a unique feature. Since the 2014 World Cup in Brazil, FIFA and the LOCs have set up a joint Sustainability Team, comprising the LOCs' sustainability teams and two full-time FIFA employees. The joint team fosters closer cooperation with the host country. Both IFs have created sustainability related awards – FIFA's Diversity Award, launched in 2016, and WS's Sustainability Award, launched in 2018^4^. FIFA's current approach to sustainability is built on the foundations laid by its CSR Department, created in 2005. WS began its sustainability journey in 2016 in order to preserve the aquatic environment on which its sport depends (source: interview with P6, 2019). Both IFs also decided not to enter partnerships with companies in certain industries (oil in the case of WS, tobacco in the case of FIFA). Finally, the leaders of both IFs (e.g., WS's CEO, FIFA's president, and secretary general) believe in the importance of sustainability actions, which may be why they have been able to adopt more strategic approaches to sustainability than many other IFs.

### Implications

Three implications can be drawn from our six case studies. First, in order to create a coherent approach to sustainability, IFs need to allocate **at least one staff member to sustainability** and build **a certain level of expertise**. Second, IFs are increasingly using **international standards**, such as ISO 20121 or the GRI, to improve the sustainability of their sports events. This is the case for four of the six IFs in our sample: The ICF and FEI have chosen GRI G4 for their sustainability goals, and FIFA and WS apply several standards, including ISO 20121, the GRI Standards, and the Greenhouse Gas Protocol. Third, the six IFs' sustainability efforts (e.g., the ICF's first Sustainability Pilot Event, in 2018) can be interpreted from **a neo-institutional perspective**. As noted in sections Approach 1: Launching Sustainability Pilot Events (e.g., the ICF) and Approach 5: Launching a Comprehensive Sustainability Strategy (e.g., FIFA and WS), external factors (e.g., expectations of the general public and athletes) strongly influenced the ICF's, FIFA's, and WS's commitment to sustainability.

### Recommendations

As sustainability actions require collaboration with almost every department in an organization and with many external stakeholders, IFs should allocate at least one full-time employee to sustainability, as is the case at FIFA and WS. Ideally, this employee should be a senior manager, so they can effectively coordinate the teams needed to implement sustainability actions.

First, an IF can encourage LOCs and national federations to embrace sustainability by including sustainability in its bid requirements and contractual documents or by providing LOCs with sustainability workshops, manuals, and/or toolkits. To do this, it must make one person in the organization responsible for sustainability. Again, the more senior this person's position, the easier it is for them to integrate sustainability into the IF's main mission, which is to organize sports events. Second, an IF may change the way it operates by adopting sustainability as one of its fundamental missions (e.g., WS)^5^ or by appointing a sustainability manager, department, and/or committee (e.g., FIFA, IIHF, WS). That is, an IF can look inward and change its structure in order to embed sustainability into its core activities (e.g., draw up a comprehensive sustainability strategy). Although both approaches are possible, the second approach is preferable.

The results of our document analysis and of the 11 in-depth interviews also enabled us to define a conceptual model that can be used to analyze the extent to which an IF has integrated sustainability into its activities. The following section describes this model, which we call the “sustainability ladder.”

## The Sustainability Ladder: Toward Integrating Sustainability in Sport Events

### Development Stage: Economic Sustainability

**Figure 2:**
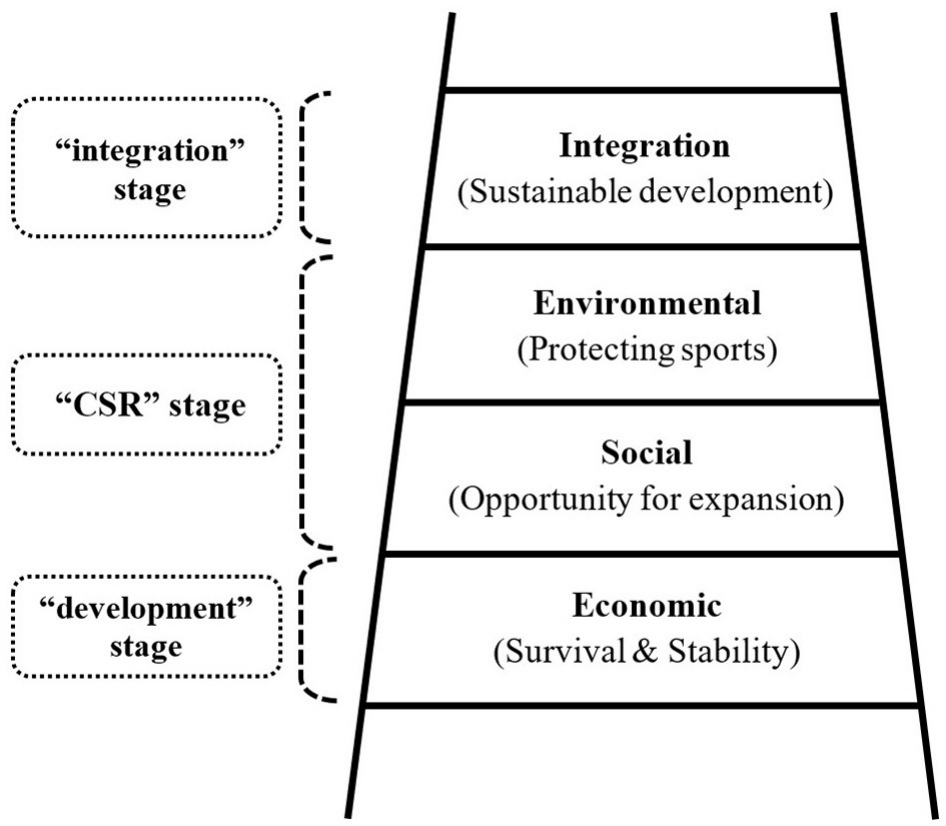
The sustainability ladder.

Given the current global economic recession, most IFs are, understandably, concerned about ensuring their sport's financial stability. As the following two quotes show, their focus tends to be on their primary mission, which is to develop their sport and thereby fulfill their *economic responsibilities* to their sport, their athletes, their member federations, their staff, and many other stakeholders (see Figure 2):

“**It's the economic dimension to sustainability**. You know, **if they do not earn money, it's not sustainable**.” (Interview with P11, 2019).

“**They need positive revenue to survive**. [….] If we are specifically talking about sports, you need sports to thrive… adapt. **Otherwise, the sport fails. You will have negative impact on your people**.” (Interview with P9, 2019).

This stage allows IFs to put themselves in a safe and stable financial position and thereby continue pursuing their numerous projects. However, it is vital for them to be wary of today's highly commercialized environment and to not forget the social and environmental dimensions of sustainability when they achieve financial stability. They can then move up to the next stage of the sustainability ladder and begin implementing social and/or environmental sustainability projects. During this first stage, they do not need to make internal and external changes to pursue sustainability initiatives.

### CSR Stage: Limited Social-Environmental Sustainability

In the second stage, IFs may use sustainability **to expand or protect their sport**^6^. They can play a leadership role both in their sport and in host communities. However, they should also start looking inward in order to integrate sustainability into their core business:

“Some football associations at the national level or UEFA, confederations… they have a certain level of development as well in this field. The IOC has certain degree of maturity as well, but the rest… you will find a few initiatives. [….] Many of federations you might talk to… they will tell you we have programs… projects on. I don't know… youth, we have a project. I don't know. If the sport is related to water, ocean plastics… we have a project there, **but sustainability in a strategic manner is more than that**. It's a way of starting, but at some point, you need to look inward… looking inward means… okay, what are our practices. It's not about projects. It's about how do we operate; how do we do business; how do we conduct our engagement with suppliers; how do we… our contracts with sponsors will be applied; how our bidding process for the events look like, so… **the integration is the key**” (Interview with P2, 2019).

Thus, whereas IFs at stage one focus on ensuring their financial survival and stability, those at stage two view sustainability as a means for expanding or protecting their sport by implementing environmental and/or social programs that are not directly related to their main events (e.g., world championships). IFs at stage two may need to make external changes and limited internal changes in order to pursue sustainability actions. For example, they can use bid requirements and/or host agreements to encourage LOCs, NFs, and host cities to plan and operate events in a more sustainable way. Internal changes may involve asking existing staff members (e.g., those in events, marketing, and/or communications departments) to devote some of their time to sustainability. In contrast with stage one, where commercial partners are very important to IFs trying to develop their sport, the main stakeholders for IFs at stage two are generally national federations, LOCs, host cities, NGOs (e.g., Peace and Sport), charities (e.g., Play for Change, SOS Kit Aid, etc.), and/or UN agencies (e.g., the UN Environment Programme, UN High Commissioner for Refugees).

The order in which an IF addresses the social and environmental dimensions of sustainability may vary according to the nature of the sport it represents (see Figure 2). For instance, IFs responsible for outdoor sports may focus on environmental sustainability as a first step and then move onto social sustainability (e.g., *via* projects promoting inclusion and diversity, gender equality, peace and reconciliation) as a second step. This is because environmental sustainability actions are more likely than social sustainability actions to have a direct impact on an outdoor sport IF's survival and stability, which are the main goals of the development stage. Thus, if an IF joins the UN's Clean Seas Campaign or Climate Change Campaign but does little to improve the sustainability of its main sport events, it cannot be considered to have gone beyond the development stage of sustainability.

### Integration Stage

To reach the integration stage, IFs must show an ethical mindset and a readiness to be “team players.” Some IFs may even need to make “sacrifices,” because this stage requires them to make internal changes. However, reaching this stage allows them to show integrity and inspirational leadership to people around the world, as well as to their sport community. IFs at stages one and two can serve as models for their athletes, members, and other stakeholders, but entering stage three shows supreme leadership, as it shows that an organization's senior executives are willing to embrace change. These changes include drawing up a new mission/policy centered round a more strategic approach to sustainability. To ensure the resulting mission, policy, and strategy do not remain empty words, an IF must appoint a sustainability manager and/or create a sustainability committee.

Major stakeholders in this stage include governments, sponsors, suppliers, manufacturers, and other commercial partners, as well as national federations, LOCs, host cities, NGOs, and UN agencies. IFs must also begin working with their stakeholders in more socially responsible ways. For example, an IF may ask its sponsors, suppliers, and/or manufacturers to provide more environmentally friendly items for its world championships (e.g., cars, food and drink, promotional and merchandising materials, souvenirs) or to use more environmentally friendly materials when producing uniforms, sports equipment, or venue materials. It could also ask them to provide items for people with disabilities or provide products that are energy and water efficient. With respect to commercial partners, such as marketing agencies and broadcasters, an IF might ask them how they would ensure a sports event is held in a sustainable way (e.g., environmentally friendly banners, pamphlets, and survey materials) or to work with the LOC to provide sign-language commentary for the deaf and/or the audio-descriptive commentary for the blind. As one of our interviewees noted, moving up to the integration stage requires major from an IF:

“I think that [international] federations resist change. If you are eager to do... one of the problems is that they identify CSR as a big change, but **CSR is not the big change**... **sustainability is the big change**” (Interview with a UN employee, 2019).

Hence, integrating sustainability into their operations requires IFs to work with a wide array of stakeholders and may entail allocating more resources and/or time to sustainability. However, some environmental programs can produce long-term cost benefits for event organizers. Despite this cost, it is essential for every sport organization to move up to this top stage for the good of their sport, of people in general, and of the planet. Table 4 summarizes the main components of the sustainability ladder.

**Table 4:** The sustainability ladder.

**Stage**	**Key determinants (values)**	**Responsibilities for whom**	**Approach**	**Key stakeholders**
**Integration**	**Sustainable development** (responsibility, leadership, integrity, dignity, pride, ethical mindset, team-player attitude, sacrifice)	Responsibilities for its sport, athletes, members, staff, and other stakeholders. Plus host communities, citizens in general, future generations	Internal changes (IFs – new mission, policy, strategy; new sustainability manager/department, committee) External changes (those listed below plus the way of working with sponsors, suppliers, manufacturers, and other commercial partners)	LOCs, NFs, host cities, governments, athletes, fans, tournament officials, media, sustainability consultancies, NGOs, UN agencies, manufacturers, other commercial partners (e.g., sponsors, suppliers, broadcasters, marketing agencies), and the IOC (Olympic IFs)
**CSR** (Social/Environmental)	**Opportunity** for expansion; **Protecting** sports and athletes (responsibility, leadership, dignity, pride, image, risk management)	Responsibilities for: its sports, athletes, members, staff, other stakeholders. Limited responsibilities for host communities, citizens in general, future generations	External changes (e.g., LOCs, NFs, host cities – bid requirements and host agreements) Limited internal changes (e.g., new tasks, given to an existing employee(s), such as events, marketing, communications staff)	LOCs, NFs, host cities, athletes, fans, tournament officials, media, NGOs (e.g., Peace and Sport), charities (e.g., Play for Change, SOS Kit Aid, etc.), UN agencies; IOC (Olympic and non-Olympic IFs)^*^
**Development** (Economic)	**Survival**; **Stability** (responsibility, leadership, safety, maintenance)	Responsibilities for: sports, athletes, members, staff, and other stakeholders	Internal/external changes for development, not sustainability	LOCs, NFs, host cities, athletes, fans, tournament officials, media, commercial partners (e.g., sponsors, suppliers, broadcasters, marketing agencies, etc.); IOC (Olympic IFs)

* *For example, eight IFs, including the International Surfing Association and World Rugby, joined the UN Environment Programme's Clean Seas campaign through the IOC in 2018*.

The sustainability ladder – which is similar to Carroll's (1991) CSR pyramid – shows how IFs can show true social responsibility and the difference between following a true sustainability strategy and implementing individual CSR projects. Many organizations used to use CSR programs as marketing tools (e.g., to enhance their reputations, improve customer loyalty, avoid being seen as irresponsible), and some for-profit companies integrated CSR into their business's strategic management with the ultimate goal of improving their financial performance (Mullen, 1997; Dean, 2003; Porter and Kramer, 2006; Walters, 2009). Even sport organizations have sometimes used CSR actions (e.g., peace, youth education, or social inclusion programs) to improve their image or to manage risks. However, once an organization enters the integration stage, sustainability is no longer about reaping direct benefits or increasing profits; it is about showing integrity and responsibility toward present and future generations. Thus, sustainability in the context of IFs can be defined as follows: “Sustainability implies a sport organization's responsibility to develop its sport for the sport itself, for stakeholders, and for the organization's survival, while minimizing the negative impact of its sport events on the community and on the environment for present and future generations.”

**Figure 3:**
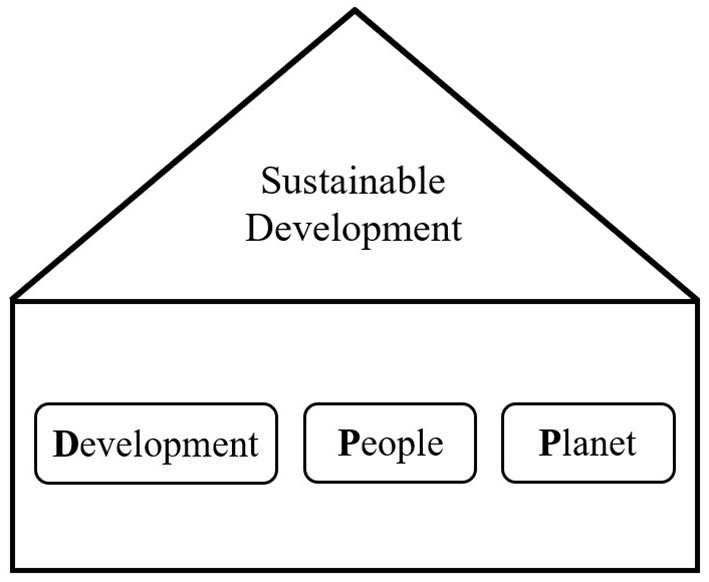
The development, people, planet dimensions of sustainability (adapted from Kaptein and Wempe, 2002; van Marrewijk, 2003, p. 101).

In summary, an IF may first need to focus on generating the revenue needed to develop its sport and ensure the continuity of its activities. Once an IF has achieved this financial security, it should move up the sustainability ladder to the CSR stage and then the integration stage. In other words, they may begin by focusing on their economic responsibilities to their immediate stakeholders, but they also have responsibilities toward people and the planet. Indeed, as Figure 3 shows, to develop sustainably, an organization must look after communities and the environment.

## Conclusions and Future Research

The present study combined François and Bayle's (2015) research model with in-depth semi-structured interviews to examine different-sized IFs approaches to social and environmental sustainability. Our findings contribute to the literature in three main ways. First, our case studies of six Olympic IFs revealed five different approaches to sustainability: (1) implementing sustainability pilot events (e.g., ICF); (2) partnering with NGOs (e.g., FISA); (3) partnering with sustainability consultancies (e.g., FEI); (4) creating a sustainability committee (e.g., IIHF); and (5) launching a comprehensive sustainability strategy with at least a full-time sustainability manager (e.g., FIFA and WS). Second, we drew up a sustainability ladder that clarifies what sustainability means to IFs by highlighting three stages in developing an integrated approach to sustainability. Third, examining IFs' approaches to sustainability through the lens of neo-institutional theory provides a better understanding of why and how IFs have begun embracing sustainability.

Although our empirical study yielded some original findings, it has some limitations. First, the results of our six case studies relate to a specific moment in time. Because IFs' sustainability strategies are continuously evolving, the situations we identified from documents published between 2015 and 2019 and interviews carried out between December 2018 and May 2019 may have changed. Second, because we interviewed only one person at each IF (except for the IIHF), we had only one account of an IF's approach to sustainability. Third, we wanted to investigate ways of measuring whether IFs' sustainability efforts have a positive impact on humanity/the planet, but we were unable to do so because most IFs' sustainability actions are still in the pilot phase (e.g., most IFs do not have a dedicated sustainability officer/manager). Nevertheless, our study provides a starting point other researchers can use to assess the progress IFs are making in this area. Demonstrating the positive impacts of sustainability actions would probably convince more sports leaders to embrace sustainability and make sports events more attractive to potential sponsors and host cities.

Our study highlighted three more avenues for future research. First, it would be interesting to extend our study to non-Olympic IFs in order to obtain a more comprehensive picture of how IFs define and approach sustainability. Indeed, our document analysis showed that many other IFs, including the International Fistball Association (IFA), World Flying Disc Federation (WFDF), and International SAMBO Federation (FIAS), have begun implementing concrete sustainability actions.

Second, it would be interesting to interview senior executives of IFs who have been very supportive of sustainability actions (e.g., Andy Hunt, CEO of WS; Beate Grupp, chair of the IIHF's Environmental & Social Activities Committee and member of the IIHF Council; Michal Buchel, CEO of the FIAS; Jörn Verleger, president of the IFA; Volker Bernardi, executive director of the WFDF). These interviews could examine these executives' academic and professional backgrounds, their motives for embracing sustainability, and their philosophies, etc., and thereby identify possible links between similarities and differences in these parameters and their federations' approaches to sustainability.

Third, future studies could investigate host cities/LOCs that have applied relatively high sustainability standards. Our interviewees mentioned several LOCs that fall into this category, including those for the 2014 World Rowing Championships in Amsterdam (Netherlands), the 2015 World Rowing Championships in Aiguebelette (France), the 2017 Nordic World Ski Championships in Lahti (Finland), the 2018 Youth Sailing World Championships in Corpus Christi (USA), and the 2019 Alpine World Ski Championships in Åre (Sweden). Studies could focus on their sustainability management systems (e.g., budget, numbers of staff and volunteers, key partners, management and evaluation tools, preparation period) and on issues such as how they coordinated with their IF on sustainability initiatives, the challenges they had to overcome, and their suggestions for future organizers. The results of such studies would also provide insights into how to measure the positive impacts of sustainability efforts.

Although some IFs may still be a long way from integrating social responsibility initiatives into their operations, we believe it is time for everyone to think about what sustainability means and how to achieve it.

## Notes

1. ISO 26000:2010 provides organizations with guidance on social responsibility and sustainable development. Available online at: www.iso.org/standard/42546.html (accessed July 5, 2021).
2. Refer to the IOC's website: Available online at: https://olympics.com/ioc/sustainability/ioc-as-leader-of-the-olympic-movement/case-studies.
3. The MSI (Maison du Sport International/International House of Sport is an office complex in Lausanne, Switzerland, that was built as a joint venture between Lausanne City Council, the Canton of Vaud, and the IOC to attract the world's international sport federations to Lausanne, the home of the IOC. This geographical proximity is intended to improve communications between these bodies. Twenty-five sport federations, ten sport organizations, and sixteen sports-related firms have taken up residence in the MSI since it opened in 2006. Available online at: www.msi-lausanne.ch (accessed September 7, 2021).
4. 11^th^ Hour Racing (2018). Corpus Christi Yacht Club Wins Sustainability Award. http://11thhourracing.org/corpus-christi-yacht-club-wins-sustainability-award/ [Accessed on August 2, 2019]; In 2016, FIFA created an annual award “to recognize an outstanding organization, initiative or football personality that stands up for diversity and anti-discrimination in football at national or international level and on a sustained basis.” Available online at: FIFA Diversity Award https://digitalhub.fifa.com/m/40d208dabfd7278a/original/zptcyr471u8ipz0fn3nq-pdf.pdf (accessed September 9, 2021).
5. World Sailing (WS) has embedded sustainability into its mission. WS announced its new vision and missions in October 2016 as follows: Vision – ‘We all work **to protect the waters of the world**'; Mission – ‘**To create a tangible sustainability program** that maximizes the positive effect that the sailing community can have on our environment' (refer to the full version of its vision and missions: https://www.sailing.org/tools/documents/SustainabilityAgenda2030-[23247].pdf).
6. The document analysis included categorizing 40 IF Sustainability Projects published by the IOC between 2016 and 2017 into five categories: (1) Environment; (2) Gender equality; (3) Inclusion; (4) Human rights; and (5) Strategy/Policy for sustainability. We divided the Environment category into the following six subgroups: (a) Water; (b) Facilities & Venues; (c) Offices; (d) Waste management; (e) Transportation; and (f) Resources reuse. The document analysis revealed two important findings. **First**, over the last decade, outdoor sport IFs (e.g., FEI, FINA, FISA, ICF, ITU, and WS) have started teaming up with other IFs, the IOC, NGOs, and/or sustainability consultancies in order to address global environmental issues. Their ultimate aim is **to protect their sports**, as these outdoor sports require unpolluted waters. Although launching sustainability campaigns (e.g., FIA – ‘Smart Cities', UIAA – ‘Respect the Mountains', World Rugby – ‘Spirit of Rugby', UCI – ‘Bike City Label', etc.) can be a good thing, it is hard to determine whether these actions are more than just communication exercises. **Second**, large numbers of IFs have included sustainability actions **in their development programs** (e.g., redistributing sports equipment, renovating sports facilities or venues, using laser pistols, rather than lead bullets, for shooting).

## Data Availability Statement

The original contributions presented in the study are included in the article/supplementary material, further inquiries can be directed to the corresponding author/s.

## Author Contributions

EB and AF contributed to conception, design of the study, and made some corrections for a better organization of the manuscript. PM organized the database, performed the statistical analysis, and wrote the first draft of the manuscript. All authors contributed to manuscript revision, read, and approved the submitted version.

## Conflict of Interest

The authors declare that the research was conducted in the absence of any commercial or financial relationships that could be construed as a potential conflict of interest.

## Publisher's Note

All claims expressed in this article are solely those of the authors and do not necessarily represent those of their affiliated organizations, or those of the publisher, the editors and the reviewers. Any product that may be evaluated in this article, or claim that may be made by its manufacturer, is not guaranteed or endorsed by the publisher.
